# Speeches, Wise and Otherwise

**Published:** 1903-04-04

**Authors:** 


### SPEECHES, WISE AND OTHERWISE.

Mr. Justice Grantham will have succeeded in adding a new terror to the life of the medical practitioner, if the latter is sufficiently impressed by the judgments he delivered during the course of the Chapman trial, on sundry other "cases" which had not been tried before him. If the practitioner wishes to do what was then inferred to be his duty, he will henceforth regard every case, chronic or otherwise, to which he may be called, as one of possible poisoning, and the anxious friends and careful attendants of his patient, as possibly or even probably, treacherous criminals. Hence he will devote less attention to curing his patient's sufferings than to assuring himself in various ways that there is no possible ground for his suspicions, and so continue, until in one way or another, the case comes to an end. It is, of course, quite sound to endeavour to remove causes rather than to treat symptoms, so the course upon which he will thus enter may be regarded as quite *fin de siècle* science. Another step he will take, when matrimonial relations apparently exist between his patient and any other person, is to make certain that they are such as would meet with the full approval of a bench of bishops; and in the alternative bring matters to the notice of his patient's parents—if he can find them. What he should do if he cannot, we have not yet been informed. Perhaps he should consult the nearest magistrate in default of direct instruction from the High Court, or at least communicate his difficulty to any lady in the neighbourhood who he may happen to know takes a special interest in her neighbours' affairs.

Seriously, indeed, it is doubtful whether *obiter dicta*, such as those in which Mr. Justice Grantham allowed himself to indulge during the progress of this case, can possibly do any good whatever. As a matter of fact it was entirely thanks to the doctor in this case, and his firm insistence on post-mortem examination, that the crime was ever discovered at all. That he did not discover for certain what was going on earlier is not to be wondered at. With a few exceptions the drugs that may be employed so as to cause lingering but eventual death, produce few if any typical symptoms, but rather give rise to a chain of events indistinguishable from those of chronic but natural disease. It is true that the real cause might be revealed by certain inconsistency or intermittence in development of the malady, but the close observation necessary to detect this would not be possible in the ordinary course of every day private practice, where for knowledge of what passes between visits, the doctor has to depend on the statements of his patient's friends. It would only be feasible, indeed, if the patient were kept under the hourly observation of a trained nurse duly provided with a note-book.] In this case, indeed, a nurse had been asked for, but the one provided was one of the "charwoman" variety, and few poisoners would be so rash as to associate any more dangerously observant person with their victim.

Where drugs are used in such quantities as to produce sudden and acutely dangerous illness, the circumstances, of course, are quite different. The symptoms then for the most part are such as to call immediate attention to their true cause, and except perhaps during the progress of certain epidemics naturally resulting in some cases of acute illness and almost sudden death, it is exceedingly improbable that any cases of poisoning, whether accidental or intentional, are ever overlooked by medical men either in this or any other civilised country. Even, however, when a doctor does suspect that his patient's symptoms are not the result of natural disease, his position is one of intense difficulty. For unless he is quite certain that the drug he suspects to be the cause of the illness is not being administered with criminal intent, he must be conscious that any action of his which may tend to reveal his suspicions, may lead to precipitate action on the part of the poisoner and the immediate death of his patient.

The remarks of Mr. Justice Grantham with reference to the duty of a medical man to interfere in matters that concern the domestic or social status of his patients scarcely call for comment. It is doubtful if there are any circumstances in which such interference would be justifiable. With his views also on cremation we are not concerned to deal: such as they are, they were duly dealt with when the bill which has just been promulgated went before the House of Commons' Committee. A doubt may be expressed as to whether the respect in which H.M. Judges are rightly held is likely to be materially increased through the delivery by any of them of opinions on matters with which they have no special knowledge and on which evidence on both sides has not been duly submitted to them for judgment.

